# Platform governance and sociological participation

**DOI:** 10.1186/s40711-023-00181-w

**Published:** 2023-03-07

**Authors:** Peng Lu, Lvjun Zhou, Xiaoguang Fan

**Affiliations:** 1 Institute of Sociology, Chinese Academy of Social Sciences, Beijing, People’s Republic of China; 2 Department of Sociology, China Women’s University, Beijing, People’s Republic of China; 3 School of Public Affairs, Zhejiang University, Hangzhou, People’s Republic of China

**Keywords:** Platform governance, Corporate social innovation, Corporate autonomy, Digital society

## Abstract

The positive and negative effects of the participation of digital platform companies in governance are a major issue of the times. Starting from the three subfields of internal governance, external governance, and co-governance, we construct an analytical framework for digital platform governance to understand the four-sided relationship among digital platform companies, the state, the market, and society. From the perspective of enterprise autonomy, we should view the effectiveness of platform company participation in social governance dialectically. The key of promoting good platform governance is to improve the external structural pressure from the state, promote the reform of the endogenous governance of enterprises, and build a sustainable architecture for co-governance. The mission of sociologists includes not only explanation but also intervention.

## Introduction

Digital platform enterprises have become an important subject in global governance and sometimes even assume “primary responsibility.” However, the social consequences caused by digital platforms are becoming increasingly mixed. Fortunately, as an important pillar of science and technology, digital platform companies have played an important role in many public affairs because of their rapid reactivity, vibrant online community, dynamic knowledge output, and capacity to improve technology applications. In China, “intelligent governance” has played a positive role in promoting the systematic architecture of national governance, achieving well-reasoned government decisions, sophisticated social governance, and efficient public services.

Concerns with the evils of capitalism and abuse of technology are also rising and have become one of the hottest social issues in the current era ever since people discovered that data, algorithms, and computing power are in the hands of digital platform companies. There is also concern over the insufficient mobilization of social forces. The rights of vulnerable groups such as elderly individuals, people with disabilities, and migrant workers are largely neglected. Regulatory problems such as algorithmic discrimination, “big data killing” (swindling regular customers by using big data), and sophisticated forms of forgery are emerging one after another. Taking the rise of “cyberpunk” culture among young people as a representative case, this trend reveals how reflection and criticism of the evils of capitalism and technology have broken through the limits of intellectual discussion to become a component of mass culture. We are in the midst of a new round of global criticism of digital technology.

That said, the involvement of digital platform companies in public governance is not completely new; their involvement was already present before COVID-19, with various scenarios in different social contexts. In China, “smart governance,” that is, digital government, smart cities, and other smart infrastructure, complemented by large amounts of data collection and application, have been actively modifying the structure of state governance, implementing more data-based policy-making processes, enhancing precision in social governance, and improving public services (He and Ma 2020; Hu and Zheng 2021; Su and Meng 2016).

In the face of these challenges in governance, it is increasingly urgent to improve digital regulations of the social field, and the issue of “platform governance” is becoming increasingly prominent. Our questions are as follows: what kind of framework can we employ to analyze the participation of digital platform companies in governance? Prior to the pandemic, what kind of “field” is constituted among different stakeholders, including digital platform companies, enterprises of other types, the state, and society? What are the characteristics of the strategies of digital platform companies in this “field,” and what are the impacts on the configuration of the field? In response to the increasing “autonomy” of digital platform companies, what position and strategy should sociology take, a discipline known for exporting “paradigms,” to promote social progress and good platform governance?

## What is the field of platform governance?

The field of platform governance is the conceptual framework we use to analyze the participatory governance of digital platform companies. The concept of a “platform” is widely applied in computer science. The Windows system, for example, is such a platform, an infrastructural meta-software that runs applied software. When the concept is used in the socioeconomic analysis, its meaning undergoes a slight change: “programmability” is no longer necessary. Although a “platform” is a formal legal concept in China, many laws, including The Law for the Protection of Consumer Rights and Interests, The Advertisement Law, Charity Law, and E-commerce Law, do not have a clear definition of the term. (Zhou and Li 2018). Combining several scholarly definitions (Gillespie 2010; Ansell and Gash 2018; Ansell and Miura 2020; Andersson Schwarz 2017), it can be said that the platform is a metaphor that resembles a market or arena in form, but in substance, it refers to a certain organization of resources: bilateral and multilateral stakeholders use platforms as intermediaries for connecting and matching with one another, thus forming a network.

A digital platform enterprise is a platform registered as an enterprise based on digitalization.^1^ This article mainly focuses on private digital platform companies, most of which are internet companies, including search engines (such as Baidu), e-commerce sites (such as Taobao), travel agents (such as Ctrip), mobile taxi booking (such as Didi), social media/entertainment (such as Tencent), and financial services (such as Alipay), which have become hubs for providing data-driven products and services.^2^

Platform companies are, first, corporations that prioritize the interests of shareholders. However, they differ from traditional corporations in that most of them, especially internet-based platform companies that provide infrastructural services, strongly influence public life. Often they need to play a role in providing, coordinating, managing, distributing, and even arbitrating public resources.^3^ That is, they serve both the public and private interests. Traditional bureaucracy and shareholder-entered corporation governance are no longer sufficient in the platform economy. Platform companies need a more open mechanism for corporate governance to deal with the interwoven relations of various external stakeholders in addition to shareholders’ interests (Fenwick et al. 2019).

Therefore, a “field” was constructed to analyze platform companies. “Field” is chosen instead of “ecology,” the term commonly used by the business world, because “field” has to do with the analytic advantage of dynamic space: it reflects a dynamic and layered structural outlook, seeing the social space, market included, as constructed through the interaction of actors (Fligstein 2001). Every “field” is governed by certain general rules or logic, driven by individual means and collective strategies; each “field” is composed of several “subfields,” which are relatively independent, running on their own secondary logic.

**Fig. 1:**
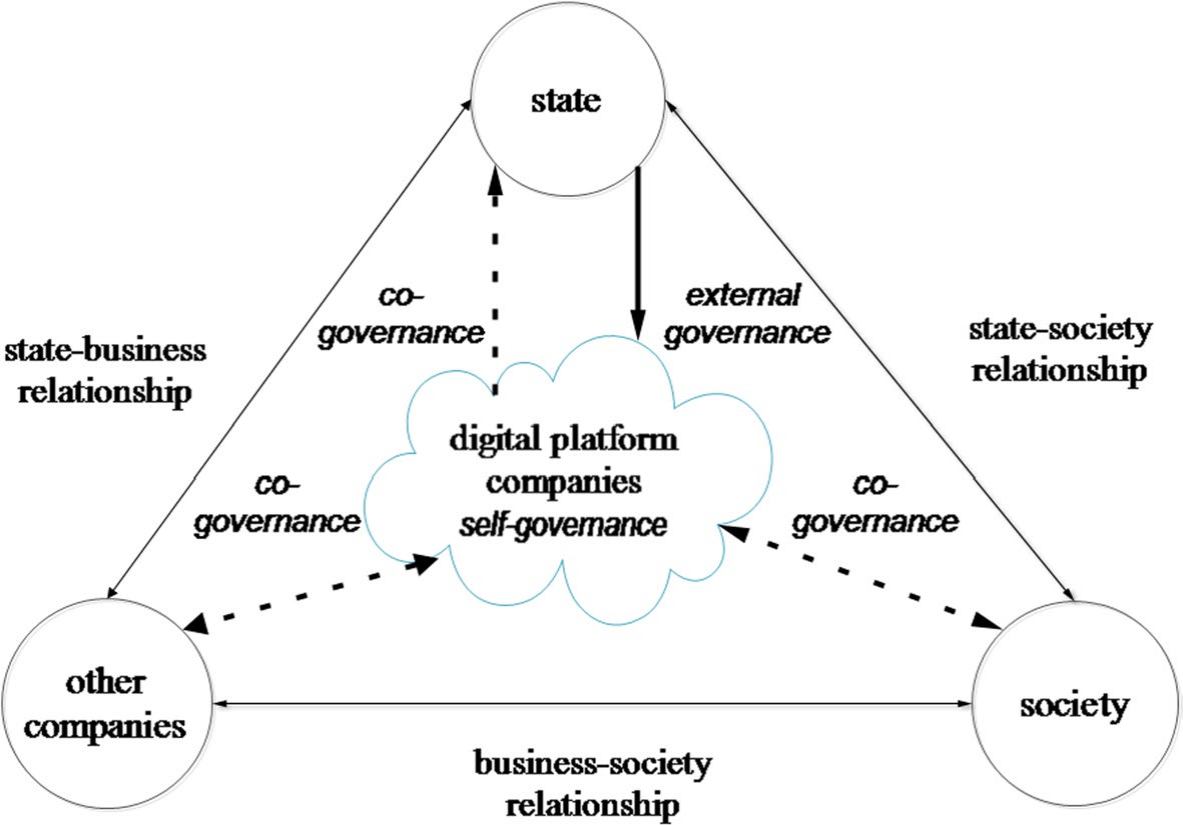
The field of digital platform governance

Some scholars think that states (including trans-governmental organizations), non-governmental organizations, and enterprises constitute the “governance triangle” for platform governance (Abbott and Snidal 2009; Gorwa 2019a). We believe that the platform governance field (Fig. 1) should be more accurately described as a space shaped by the quadrilateral relationships of the state, digital platform companies, other enterprises, and society (including customers and social institutions); the digital platform company is not only the “governor” but also the governed (Gillespie 2017), as well as the intermediary for “state-market-society.” There is a vertical level of formal systematic regulations (international treaties, national legislation, industry norms, user agreements, and platform conventions) in platform governance, but it also shows the characteristics of a horizontal network. Various informal governance systems have a ubiquitous influence on agenda setting, system diffusion, and policy implementation. Gorwa (2019a, b) divides platform governance into three modes: self-governance, external governance, and co-governance. In our view, self-governance and external governance are “subfields” of different levels, while co-governance is the “meta-field” of digital platform governance; the three coexist and converge into one another.

*Self-governance* The narrow sense of “platform governance” in some management literature only refers to the “corporate governance” of platform companies, including the upgrading of technology or products, the establishment of a more open and transparent corporate governance structure, the improvement of algorithms, and the formulation of platform agreements. However, many of the changes implemented within these companies will also involve how the platform companies standardize their B-end (suppliers and advertisers) and C-end (users) ecologies, which is a type of “governance by the platform,” merging with the co-governance. The platform can fully rely on enterprise rules, digital technology, and third-party institutions to build a hierarchical control system “across enterprise boundaries” or outside the enterprise (Zhao and Han 2021).

*External governance* It is the governance of the state.^4^ The “platform governance” discussed in political science literature refers to this second definition. Large technology companies are increasingly involved in governance, but at the same time, due to numerous public scandals, such as large-scale data disclosure, monopolies on the platform industry, and increasing public attention to polarization, false information (such as “fake news”), and algorithm colonization, the supervision of these platform companies has become the focus of many critical studies. In the Western context, avoiding “platform capitalism” (Srnicek 2017), “surveillance capitalism” (Zuboff 2015), and “data capitalism” (West 2019) and protecting the “freedom of speech” and democratic governance (Suzor 2019; Vaidhyanathan 2018), as well as curbing the impact of transnational technology companies on global governance (Gorwa 2019b), are hot topics in both the policy-making world and academia. In China, the main concern includes the challenges brought about by the participation of large technology companies in policy-making, which may cause systematic risks, endanger political security (Fan 2019), and weaken the local government’s grip on the economy (Zhang 2021).

*Co-governance* Here, we explain the relationship among digital platform companies, other types of companies, the state, and society. The logic of domination in this field is to pursue governance efficiency (legitimacy). There are two ways digital platform companies can participate in governance: (1) participate in one part of the national governance architecture (P2G). For example, they can participate in governance indirectly as suppliers of “e-government,” mainly providing “technology.” (2) What truly triggers wide discussion is when digital platform companies directly become a main body of public governance. Some of them participate in civil society governance through “users” management (P2C);^5^ some participate in the shaping of the relationship between government and business, corporations and society through interacting with other companies in the “market” (P2B), and even exert a lasting impact on the social structure in a broader sense. Klonick (2017) believes that large platform companies have become “private governors” by creating rules and making decisions in many fields related to basic civil rights. In fact, governments of all countries regard the internal corporate structure of the platform as a tool to realize their preferred policy consequences, thus forming a “mixed public–private governance structure,” which can even spread their domestic laws to global users without much effort through the rules of platform companies, such as user service agreements (Bloch-Wehba 2019).

This governance model has triggered a series of urgent legal issues, calling attention to strengthening “external governance.” What we want to emphasize, however, is that the relative autonomy of platform companies in co-governance should not only be regarded as the object of state regulation. Although governance is often understood as state capacity (Fukuyama 2013), governance is not only about building state capacity but also about rule-making and implementing a complex interactive network concerning different actors. As Stoker (2018) pointed out, the consequence of governance is the creation of order and collective action. Modern governance theory has already broken through the narrative of “state centrism,” emphasizing collaborative governance involving the participation of corporations, social organizations, and other forces (Rosenau et al. 1992). In essence, collaborative governance is consistent with co-governance, which comes from Elinor Ostrom’s polycentric governance theory (Ostrom 2005). Ostrom’s theory emphasizes that the government, society, and the private sector communicate with one another toward a goal achievable only by joint forces. Collaborative governance has received great scholarly attention in recent years and has merged with studies on the coproduction of public goods; it is the subject of many emerging studies in the field of policy research and public administration (Brudney 2020). Research on platform governance should pay more attention to the interactions among multiple stakeholders, such as the state, enterprises, corporate foundations, civil society organizations, and public media.

Just as the question “what is governance” (Fukuyama 2016) caused a series of debates around conceptual confusion, platform governance is also a messy concept.^6^ Using the analytical concept of the “field,” the hierarchical division of external governance, self-governance, and co-governance will not only help us clarify the relations among the main stakeholders of digital platform companies, states, other types of companies, and society on the empirical level but also enable us to fit different “platform governance” concepts into a unified analytical framework.

This analytic framework can also analyze any phenomenon of corporations participating in governance in general after a slight modification. The role of large companies in nation-states and global governance is gaining increasing importance, which is not unique to digital platform companies. However, the difference between digital platform companies and real estate, energy, finance, and other industries with corporate giants is that the state cannot completely replace technology companies; instead, it relies on them. Particularly in today’s world, digital technology and algorithms are inseparable, so the literature on “platform governance” is often combined with studies of the impact of algorithms on governance and the governance of algorithms (Doneda and Almeida 2016; Musiani 2013). It is precisely the technical characteristics, which resemble a “black box,” that make it so unpredictable, arousing fear in the mind of the public. In fact, in digital governance, technology itself becomes an independent variable. Technologists tend to believe that by full application of technical means, many problems in platform governance can be solved automatically. For example, to reduce the risk of passengers facing potential danger, drivers are required to register with face recognition software before receiving orders. However, the reflection on the role of “technology” in the “state-market-society” relationship, especially the vigilance against “technology evildoing,” has always been a long-term question of “technology/knowledge sociology” (Foucault 1977; Smith and Marx 1994). Sociology has unique advantages over research on the governance of digital platform companies. Sociological literature rarely regards state discipline or the “managerial revolution” of companies as a panacea for technological evils; in contrast, sociology urges people to be vigilant of the collusion between power and knowledge/technology and use various “informal” strategies to break the domination of various “technology-power” governmentalities.

Therefore, sociological research on the structure of platform governance should not only seek to understand the complex power relations and governance structure in today's “platform society” (Van Dijck et al. 2018) to hold a dialog with the rich research tradition of power and market structures in sociology (Domhoff 2006; Fligstein 2001; Granovetter 1984; Mills 1956; Scott 1997; Swedberg 1991; Useem 1984) but also ask about the governance efficiency as well as the consequences of the social policy of this power structure. Likewise, the question of how digital platforms can be more beneficial to the public rather than a select minority should be combined with the long-term concern of sociology for social equality, especially the digital divide (Ignatow and Robinson 2017). As we will elaborate in the fourth part of this paper, sociological intervention is a crucial means for good platform governance.

Bourdieu uses two metaphors to elucidate the concept of “field,” that is, it is both a “force field” and a “playing field.” (Bourdieu and Wacquant 1992) The “force field” reflects the pattern of objective relations among various social positions in the field. The “playing field” reminds us of the role of subjective cognition and strategic action in field operations. The next question is, what kind of judgment can we draw on the big picture of China's platform governance field against this analytical framework? What are the characteristics of the action strategies of platform companies in this field? What impact has it had on the platform governance pattern? The next two sections will answer these questions.

## The general structure and enterprise strategy of the platform governance field

As a leading nation in applied digital technology, China has formed a “platform governance field” with “asymmetric interdependence” (Zhang 2019) between the state and digital platform companies. In Bourdieusian terms, the state and platform companies constitute the dominant group in the field of platform governance, within which the state is the dominator and companies are dominated.

From the external governance perspective, increasingly strict regulation of digital platform companies has become a trend in all of the world’s leading economies. China has promulgated a series of market regulations in price mechanisms, payment mechanisms, consumer rights protection, reasonable taxation, and fair competition. Compared with the EU's controversially harsh governance, the Chinese state is tolerant of corporations in regard to issues related to public policies, such as privacy and ethics. Although some people are worried about the “Leviathan encountering the Unicorn” (Fan 2018), others believe that this is one of the reasons why the Chinese market is more innovative and dynamic. With the rapid development of technology and the continuous expansion of applications, the public’s disputes about privacy, fairness, responsibility, security, and application boundaries are also growing in China. The state must actively seek a dynamic balance between risk prevention and industrial upgrades. In 2020–2021, with the introduction of a series of regulations and policies to prevent the disorderly expansion of capital, promote internet security and bring into effect the Data Security Law and the Personal Information Protection Law, China's supervision of platform enterprises entered a new phase.

The “external governance” of the state is not to control the internet but to better regulate and develop the platform economy. According to the *Outline of the 14th Five-Year Plan (2021–2025) for National Economic and Social Development and Vision 2035 of the People's Republic of China*, “regulatory overreach should be rolled back where it hurts business dynamism, while regulation will be strengthened wherever necessary in our effort to create an open, safe, and healthy rules-based digital ecosystem.” Strengthening external governance should further improve internal governance within platform enterprises to create a healthier new pattern of common governance. From this standpoint, we should realize that just as the state has its “relative autonomy” (Evans et al. 1985), corporations also have their own relative autonomy. To a certain extent, the theory of “national autonomy” emphasizes that the state is not a tool of (bourgeois) rule; the proposition of enterprise autonomy requires us to regard the enterprise as an organizational entity with independent capability, logic, and mechanisms. Corporations are not only the objects tamed by the (authoritarian) state but also the “accomplices” of the “Digital Leviathan” or the tools of “state capitalism.” State-centered governance theory is used to explain the formation and diffusion of governance structure from the perspective of the state as the decisive actor. It lacks the “market perspective” that sees the internal vitality and logic of corporations in national governance from the perspective of the corporation as an agency (Lu and Liu 2021). Only when platform enterprises sincerely combine national regulation and social expectations with their own logic can they truly promote the sustainable development of good platform governance.

Taking platform companies as the center of analysis, we find that they have taken many voluntary measures in both self-governance and co-governance. In self-governance in recent years, an increasing number of Chinese platform companies have been breaking the barriers of traditional “corporate governance” and moving toward an open governance structure. They have set up institutions such as research institutes (corporate think tanks) and ethics committees, established corporate foundations, and worked with third-party institutions, social organizations, and other partners; enterprises’ internal governance strategy becomes more credible through these efforts. Some enterprise founders personally participated in and designed enterprises’ environmental, social, and governance (ESG) projects, which brought much new potential energy to the enterprise and impacted the entire industry.

How do platform companies work with the state and form a common governance structure? To answer this question, we take the performance of platform companies in the early stage of the epidemic as an example for analysis. The epidemic was an external shock to platform governance, ironically creating an opportunity for platform enterprises to compete with other entities. Through Baidu search, WeChat search, and Bing search, we collected examples of the “top 20” internet enterprises showcasing the power of science and technology support. We summarized 162 application scenarios and organized them into ten categories. The “top 20” enterprises come from the list of “Top 100 Chinese Internet Enterprises,” jointly released in 2019 by the Internet Society of China and the Ministry of Industry and Information Technology. Our data collection ended on April 6, 2020, and we modified the categorization by refining the 162 scenarios into two categories. The first type is internal governance, including the initiatives taken by the platform that affect users and business partners. The second type is co-governance. The cooperation partners include foreign governmental organizations, domestic governments at all levels, public institutions, including medical institutions and scientific research centers, and official charitable organizations.

**Fig. 2:**
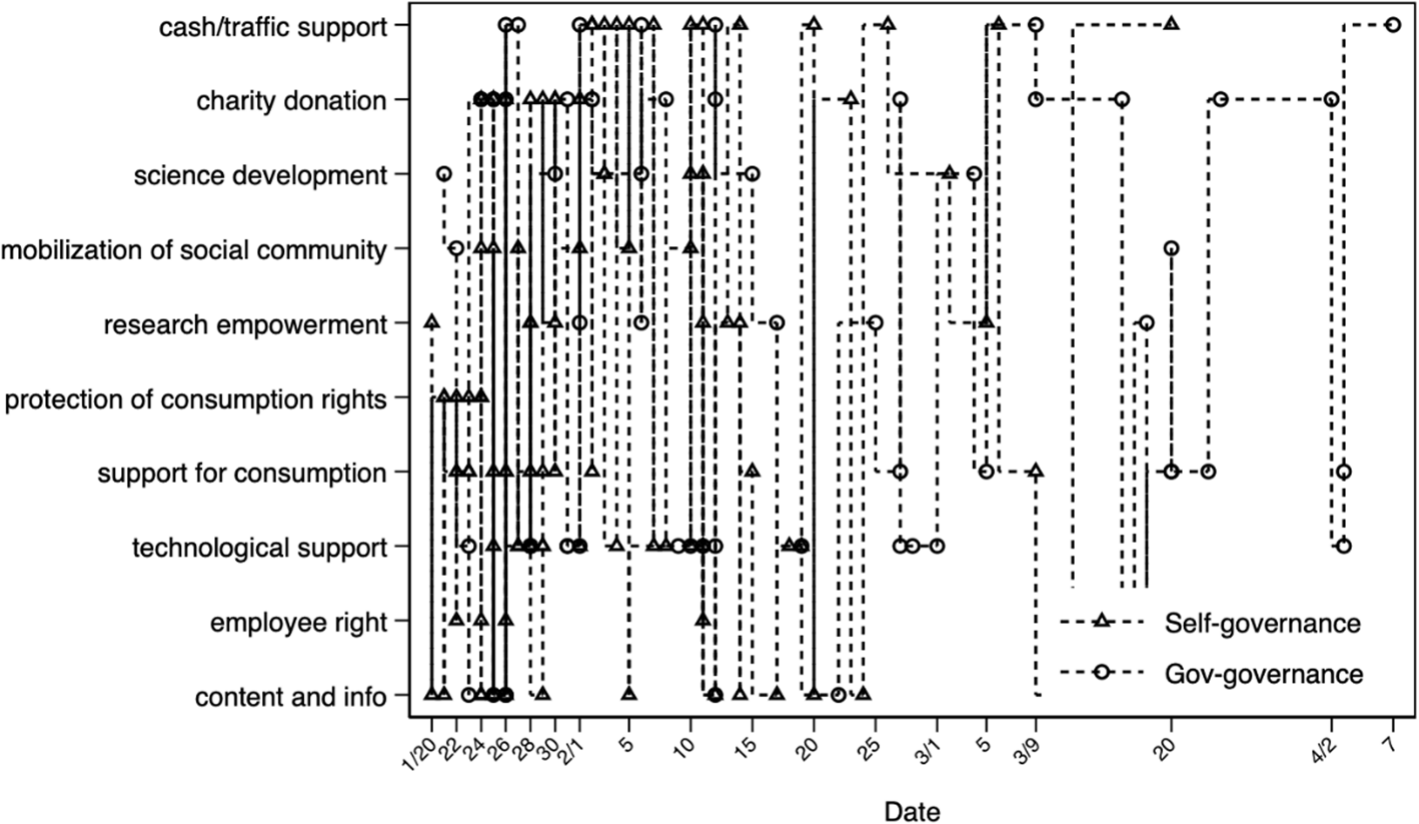
Types of initiatives of top 20 Chinese digital platform companies over time

Figure 2 shows how the types of action on the platform companies change over time. On the evening of January 20, Zhong Nanshan’s interview with Bai Yansong was regarded as a “turning point” in the development of the epidemic. After the interview, the epidemic truly became a national event. In Fig. 2, the horizontal axis is the timeline, and the vertical axis is the scene type. Each square or circle represents a corresponding scenario of internal governance or co-governance. The solid and dashed lines in the figure have no substantive meaning but are convenient for the reader to refer to the positions on the horizontal and vertical axes.

In the period that began on January 20, the internal governance of the platform was the most concentrated in the early stage; over time, an increasing number of joint governance actions were taken in cooperation with the government, hospitals, and scientific research institutions. The former mainly included consumer rights protection, content information governance, employee protection, community mobilization on the platform, and consumption support initiated by the platform; the latter mainly included technical support, scientific and technological development, financial/flow support for cooperation with the government, research empowerment, and consumption support for cooperation with the government.

In the early stage, there were two reasons for the uptick in the internal governance of the platform: on the one hand, SARS-CoV-2 was a brand-new virus, and some solutions used by past platform governance could not be directly applied. In addition, most platforms had not accumulated policies for the public health field, so much work needed to be started from scratch and time spent matching new partners. For example, the epidemic prompted some platform enterprises to cooperate with the health department for the first time to obtain distribution information for fever clinics nationwide to launch on a map. On the other hand, the products and services first launched by platform companies during the epidemic are inseparable from accumulated advantages over time, displaying clear path dependency. The first response of many digital platform companies was to quickly launch “emergent” measures based on their existing products and service advantages.

Take the governance of content information as an example. Whether refuting rumors or distributing authoritative information on preventing and treating COVID-19, the ultimate determinant is the platform’s investment in “knowledge/content” governance. Enterprises that invest in medical and health knowledge and have a strong “background” and good platform governance are more likely to attract more views during the epidemic. In this regard, the gap between different platform companies was obvious. Some platforms could not launch their own products and services until ten days later. As early as January 21, “Dr. Clove,” an online health consultation platform, launched a map of the epidemic and real-time broadcast on the WeChat official account. In fact, many of the actions of Dr. Clove were just to integrate the authoritative information released by the government. However, with the huge number of users accumulated by its previous original content, the credibility gained by past platform governance, and the technology for straightforward, real-time visualizations, it exceeded the popularity of the reporting by many official media outlets within a short period; it was so popular that even People’s Daily cooperated with it.

We take the fight against the overpricing of medical masks as an example. Almost every e-commerce platform imaginable announced a policy of “no price gouging.” However, was there truly no price increase, or were these statements just a gesture of goodwill of the enterprise? At that moment, the ability to manage suppliers helped the enterprise stand out. Some platforms have obvious advantages, while some e-commerce companies can get the attention of the platform, but the actual effect on sales is not ideal. These platforms could only provide special subsidies to the masks produced by self-employed channels to ensure limits on price gouging.

The same was true for platform companies that mobilized communities to participate in governance. During the epidemic, offline community mobilization was subject to various constraints. Whoever could fully mobilize the online community could win the first battle. Due to accumulated experience with digital rural governance, Tencent’s information service platform “Wecounty” started monitoring public opinion on epidemic prevention in villages from January 22. It urgently increased the number of messages issued by the village committee to the villagers in the notice template and promptly transmitted authoritative epidemic information to rural people through “today’s hot news” and WeChat official accounts. In addition, it provided positive guidance against “excessive” lock-down behaviors in the village, such as road closures and intimidation or violence. For example, the Transfar Group built a nationwide green logistics channel for anti-epidemic materials, providing free transportation connections and warehousing and material transfer services. In less than three days, it received nearly 200 demands for transportation at full capacity destined for Wuhan and delivered the materials to Hubei. Transfar could deliver all these services because they developed an online community app called “Transfar Comfort Station,” where truck drivers helped each other and created a community that values public welfare. During the epidemic, 22,000 community netizens responded positively to the app, inseparable from community accumulation, cultural construction, and even online party building.

Figure 2 also shows that the same application scenario has a certain effect of “innovation–learning/catching up–diffusion–anchoring” among peers. The “anchoring effect” was most obvious in the donations made by platform companies, not only in the number of donations but also in donation preference (Li et al. 2020). We take consumer rights protection as an example. On January 21, Ctrip was the first to announce that users who were diagnosed with COVID-19 or who were quarantined and urged to return by the airport (port), as well as fellow travelers who were close contacts, were allowed to cancel all bookings on the Ctrip platform for free. Around the time Ctrip made that statement, most major online travel agents also launched similar policies. Other measures also had the same characteristics, including the efforts of e-commerce platforms to combat price gouging and social media platforms to refute rumors and promote contactless services. On the one hand, this brought about a rapid “institutional learning and diffusion” effect; on the other hand, it displayed high homogeneity.

As the epidemic escalated, there were increasing scenarios for co-governance. Many cooperations resulted from government requirements (such as issuing purchase coupons) and from active matching by platform companies. For example, on February 10, at the press conference of the Joint Prevention and Control Mechanism of the State Council to cope with the outbreak of COVID-19, Yueliang Chen, the director of the Department of Community Governance at the Ministry of Civil Affairs, said: “Technical support is very important. I ask those large internet companies, such as Tencent and Alibaba, to develop community public software and provide it to social workers. A useful public welfare software is more valuable than donating one billion yuan.” Soon after, Alibaba launched a free smart community epidemic prevention and control applet.

Because the “anti-epidemic” products and services of the platform companies met the demands of governance, we witnessed the diffusion of large-scale technology applications under strong government support. A notable feature of this process was that in the cities where enterprises are based, the local government took the lead in supporting platform programs, which then diffused nationwide. The application scenarios included AI temperature measurement and agricultural produce sales through live streaming. This model had existed before, but the COVID-19 outbreak accelerated the diffusion. The most typical example was the rapid spread of the “Health Code” from the local to the national level. Alibaba and Tencent took the lead in launching their own health code products, first in Hangzhou and Shenzhen, respectively, where their headquarters are located. Taking Alipay’s health code as an example, the process from local cities to nationwide application of health codes only took seven days. According to media reports, the Yuhang District Government of Hangzhou City first adopted Alibaba’s health codes on February 9, and Hangzhou City and all of Zhejiang Province^7^ followed. On February 16, the E-government Bureau of the General Office of the Chinese central government gave the “green light” to Alibaba to speed up the dissemination of these codes nationwide to 100-plus cities. It was not the only app that spread quickly; many similar products in other Chinese cities followed a similar trajectory. Fast diffusion was not an exclusive phenomenon for Alipay products; similar products developed by other companies, such as “Suishen Code” in Shanghai, “Suikang Code” in Guangzhou, and “Jiankang Bao” in Beijing, also rapidly spread. At the same time, the infrastructural layer of health codes spread to implementation in various scenarios, from transportation, returning to work, catering, and returning to school. In fact, platform-generated health codes became normalized and formed a huge “code industry.” Some people even predicted that these enterprise-initiated health codes might become real code through application across scenarios, and implementation would hardly encounter any major public disputes (unlike privacy issues), like many other policies during the COVID-19 outbreak.

**Fig. 3:**
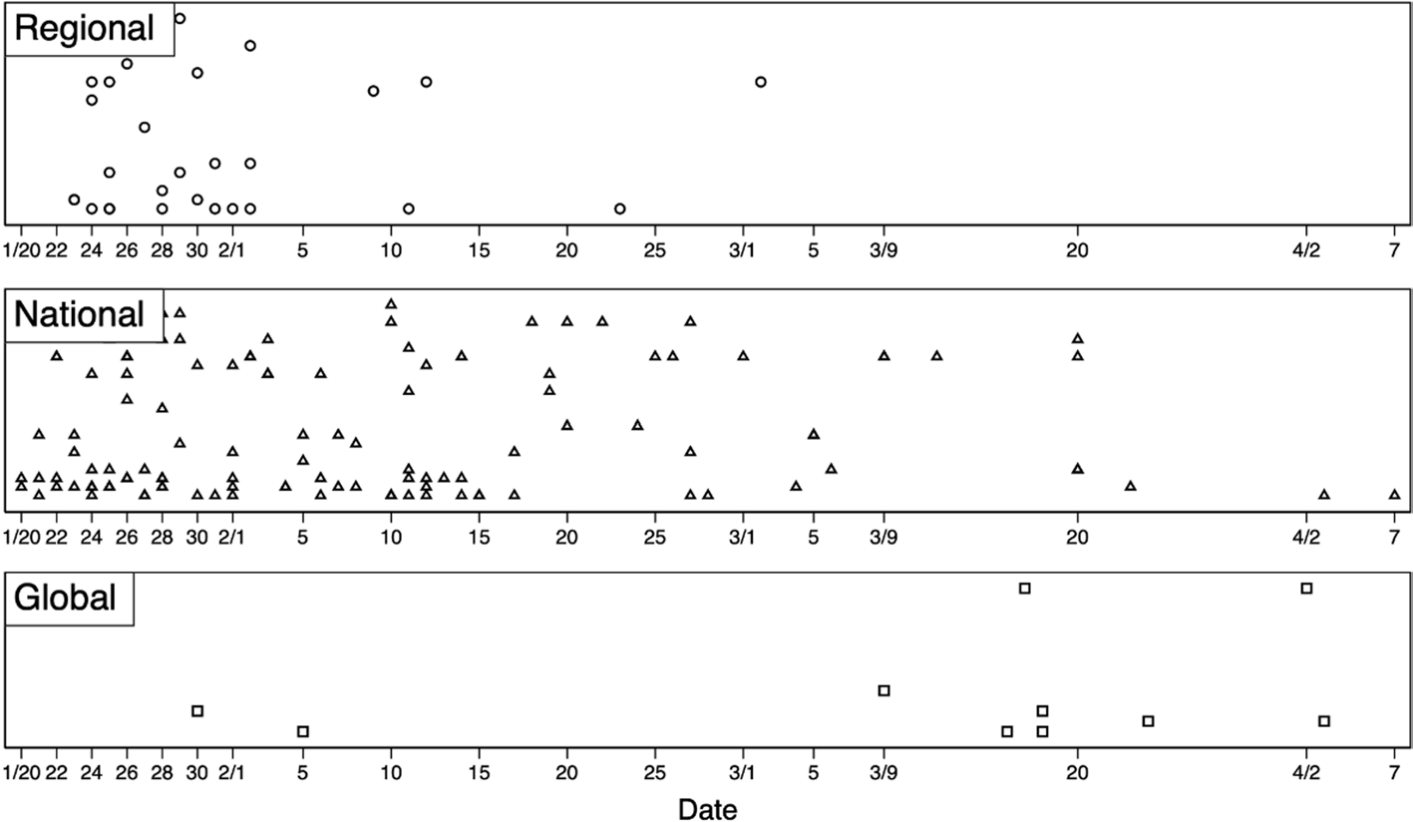
Trend of the geographic coverage of new scenario beneficiaries by day

Figure 3 clearly shows a trend in which the beneficiaries of the application scenario moved from regional to national and global. The position corresponding to the vertical axis in the figure is consistent with that in Fig. 2. Due to limited space, it cannot be expanded in detail. The trend was certainly related to changes in the epidemic (for example, the outbreak of the overseas epidemic stimulated some Chinese platform companies to invest in global competition with anti-epidemic measures). However, as mentioned before, it also resulted from the co-governance between the platforms and the government. An increasing number of technologies found national partners, and increasing technological achievements were quickly implemented in various scenarios by the model in which the “government shapes the market.” With the escalation of the epidemic, not only have the types of scenarios for platform companies to participate in governance become more diverse but the scale covered and the depth involved have all been improved, contributing to better governance performance.

Overall, the platform governance structure where government and business intersect is jointly shaped by the state and enterprises. The state is the leading party and rule maker in this process, but enterprises are not completely passive recipients; instead, they can and should actively pursue good platform governance. Platform governance between the state and enterprises is not a zero-sum game: the main demands of the state include legitimacy and economic development; the demands of platform companies also include development (profitability) and legitimacy. There is no substantive contradiction in it, which allows for coexistence. This overlap in goals explains the internal logic of why the state and enterprises have more affinity on some governance issues than the general contours of society as expressed through demands. It is a microcosm of the broader relationship between politics and business.

“Data governance” involves the government, internet enterprises, technology communities, non-governmental organizations, citizens, and other stakeholders. It is no longer a tool for enterprise value or government management but an infrastructure for forging interdependence among data stakeholders and developing the value of data itself (Xu 2021). Currently, national governance extends from physical to virtual space, which greatly increases the difficulty of governance (Lu 2019). The mismatch between the traditional governance paradigm of the state and the economic characteristics of internet platforms led to the failure of state governance from time to time (Wang and Zhou 2019). The construction of a digital government with the power of digital technology can cope with this failure to a certain extent. In addition, China’s soft power in the global digital platform regulatory system is relatively weak; as China’s internet enterprises make increasing efforts to “go overseas,” it is inevitable that these enterprises will be involved in global governance. Against this background, the country strongly demands digital tech companies to support state governance, and digital platform companies also have more room for development.

## Positive and negative efforts and sustainability, good governance of platform companies, and participation in governance

Digital technology plays an important role in common governance, and digital platform companies have become one of the multiple collaborative governance entities that cooperate with the state in supervising users. In the past two years, many documents have emphasized the general direction of “technologically supported” social governance. For example, the 14th Five-Year Plan, issued by the Chinese central government on March 13, 2021, comprehensively discusses “accelerating digital development and building a digital China” in Chapter 5 and proposes “accelerating the construction of a digital economy, a digital society, and a digital government, and driving the transformation of production, living, and governance modes through digital transformation as a whole”; the same document proposes “encouraging social forces to participate in the “Internet+ public service” and to innovate service modes and products.” In the section “strengthening and innovating social governance,” it is clearly stated that it is necessary to “facilitate and regulate the channels for market participants, new social strata, social workers, and volunteers to participate in social governance.” Another document, The Opinions of the CPC Central Committee and the State Council on Strengthening the Modernization of the Grassroots Governance System and Governance Capacity, issued on July 11, 2021, puts forward clear requirements for “empowering the building of grassroots-knowledgeable governance capacity” and implemented the agenda of “internet + grassroots governance.” These policies improved digital availability and community surveillance of grassroots governance.

At the practical level, He and Ma (2020) illustrate how an internet company in China promoted mobile payments in medical insurance. They find that when corporate strategy and public interest are organically combined, private companies can also facilitate policy innovation in the public sector. In fact, platform companies have entered many fields previously dominated by offline modes. For example, the live-streaming economy went viral during the epidemic, and there was an upsurge of “county magistrates live-streaming.” Although spontaneous acts of a similar character occasionally existed, if the external impact of the epidemic had not provided the legitimacy and urgency for government officials to enter live-streaming rooms to promote local produce and products, this phenomenal scenario might not spread so quickly.

On the other hand, although we can see that the expansion of digital technology has brought some positive governance efficiency, the pressure on external platform governance will not be eliminated in the long run, whether at home or abroad. In fact, the spread of digital technology has also brought some negative effects. For example, data privacy has received unprecedented attention, and some legal and institutional gaps have to be filled to provide a clearer definition of user rights. The disorderly expansion of capital leads to the risk of market monopolies. Enterprises operating outside the market rules need to follow more social norms and bear more social responsibilities; the risks brought by data transactions have surged, and national data security supervision has intensified unprecedentedly.

In addition to the phenomena that have been closely examined and discussed from the perspective of the “state–market” relationship, another phenomenon that has received less attention is “selective governance.” In areas that both the enterprises and the state emphasize at a specific time, government–enterprise cooperation is relatively smooth, and governance efficiency is significantly improved; on the other hand, areas that are neglected by both the state and enterprises will become the weak side of governance, while areas that are of interest to only one of the actors may become unbalanced. For example, during the epidemic, the paper certificates in the hands of residents were in sharp contrast to the newly instituted facial recognition access device at the door, and the so-called informatization in many places became a synonym for the extra input of information. The health codes in various places were not common to other places, and even a case such as “adding codes to the codes” appeared. What was embarrassing about these new technologies was that in many “social” scenarios, due to poor coverage, limited daily active users, and different standards, the traditional mobilization methods of household visits and monitoring must be adopted to meet treatment requirements (such as “full coverage”) during the “wartime” situation of the epidemic.

This coexistence of “advanced” technical equipment and “backward” data collection tactics reflected the reality of the conditions of limited sources and poor quality of data collection tools, insufficient circulation of information, and serious data isolation accumulated over a long time (China’s Institute of information and communications 2020). The lack of “labeling” and the “social situation” also led to the serious homogenization of scientific and technological products related to the epidemic. For example, browsing through the “AI-supported COVID-19 epidemic prevention and control information platform,” which was managed by the Department of Science and Technology at the Ministry of Industry and Information Technology and constructed by China's Artificial Intelligence Industry Alliance, we found that although there were more than 500 AI products and services related to the epidemic, the categories mainly concentrated on intelligent identification (temperature measurement), intelligent medical systems, intelligent robots (including UAVs), and types of hardware and tools such as emergency dispatching and online office platforms.

One consequence of this selective governance was the “crossing problem that mingled hope and fear” encountered by platform companies in past periods. In the first half of 2020, we witnessed many achievements in technological support for combating COVID-19, and therefore, the “excitement curve” rose; in the second half of the year, negative problems such as the excessive collection of data, proliferated scamming, misuse of facial recognition, and discriminatory pricing based on big data intensified people’s worry about “technology doing evil,” and therefore, the “anxiety curve” began to rise. This intersection reflects the ongoing tension between technology and society in human history and the weakness of the platform governance field in the past. Platform companies took the lead in some fields and achieved good results in governance, while governance failure occurred in other fields.

To balance the pros and cons of platform companies and promote sustainable platform governance, it is not enough just to provide modified technical solutions or more effective protocols (Liang and Zheng 2020); the institution in which technical applications are embedded must change as well. Corresponding to the three levels of “platform governance,” this system also includes three levels, namely the external governance system, internal governance system, and common governance system. Policy-makers have paid attention to external governance and common governance system, which this article discussed several times. However, a problem often overlooked is how these regulatory requirements from outside the enterprise can become the internal values guiding enterprise behavior to achieve enduring good platform governance.

From the perspective of laws, regulations, documents, and practice, platform companies have become members of the diversified collaborative governance subject under the state. However, many people question the motivation of enterprises seeking participation in sociopolitical affairs. Most of the time, enterprises are still confined to “pursuing profits.” Enterprises do not exist as an independent variable in social governance; the social influence and autonomy of enterprises have been ignored by some current theories. However, according to the theory of corporate citizenship, which has been popular in the field of “enterprise–society” relations for more than a decade, enterprises are also citizens rather than simply “like” citizens. They can participate in society and governance out of their own motivation rather than the external pressure, like citizens and natural persons (Matten and Crane 2005; Moon et al. 2005).

We should not limit our understanding of the enterprise's active participation in governance as “profit-seeking.” As pointed out at the beginning of the article, an important feature of platform companies is that they are born to have strong governance attributes. Today, they are facing an increasing number of new governance problems, including the legal and rule-binding acquisition of data, the definition of legal liability for infringements on the platform, algorithm values, and platform monopolies. “Compared with traditional issues such as infringement and counterfeiting, false publicity and bad information, these governance issues can get on the sensitive nerves of the public, which further increases the complexity of platform governance” (China Academy of Information and Communications Technology 2019). These disputes are directly related to the legitimacy of the core business model of platform companies. The lack of prudence puts them in the middle of national regulation and public opinion, which may endanger the very survival of the products or even the platform itself. Therefore, legitimacy is not only the concern of the state as the main body of governance but also that of the enterprises, either as the ruling body in the market or governance. Enterprises must act on their own instead of waiting for the government to act.

Companies’ self-governance is an important means to obtain legitimacy. Studying the self-regulation of internet companies in the USA, scholars find that many measures of these companies are actually preemptive solutions to avoid government intervention (Newman and Bach 2004). China’s platform companies also have similar considerations. Some have tried influencing state legislation, government decision-making, or public opinion through various lobbying channels to insert corporate rules into industry norms and laws (Huang and Chen 2020; Kennedy 2008). Others have found that Chinese internet companies have played an important role in the co-governance projects that involve different levels and departments of the state through a series of strategies (Jing and Li 2018). Even if the ultimate result of these actions is enhancing these companies’ economic performance (yet the causal mechanism in this is not so easy to ascertain), the primary intention of these actions is to obtain legitimacy.

Corporate social responsibility (CSR) should also be seen in this way. There are many external “obstacles” to platform governance from the perspective of platform companies. They include the local government’s lack of sufficient ability to cope with social issues caused by the platform economy and the low digital literacy of officials, which are reflected in the chaotic platform governance of ride-sharing (Li and Fu 2019; Wang and Zhou 2019). For instance, insufficient digitalization of some companies, customers’ lack of trust in digital platforms, limited applications to social scenarios, and even ideological resistance to technological applications can all impact the capacity of enterprises for platform governance. CSR projects can provide channels for the government and the public to understand company decision-making processes and governance mechanisms through transparent measures and mobilize NGOs and users to become involved in platform governance.

There will still be doubts about many actions implemented in the name of CSR, which is perceived as serving the “public relations campaign” of the company to a large extent. These concerns are not groundless because it is difficult to distinguish political from social motives in the public behavior of companies (Moon and Knudsen 2018). Even empowerment for the disadvantaged is motivated by profit-seeking, increasing user trust, and expanding the market (Zheng 2019). However, as some researchers have pointed out, based on the study of Shell’s CSR Report, on the one hand, Shell’s commitment to sustainable development aims to establish its hegemony in the market by integrating the concept of sustainable development into the business’ discursive system to meet the challenges of environmentalists; on the other hand, the company’s concessions and embrace of the commitment to “sustainable development” have also changed its practice, making Shell a model of progress in the field of environmental protection. Its new practice in sustainable development is even more radical than its public statements in many aspects. Moreover, its support for the Kyoto Protocol has broken the united front of the energy industry in facing global warming and put pressure on its competitors (Livesey 2002). In other words, the impact of these actions is not entirely under the control of the companies. The companies’ embrace of “good governance” will, in turn, tie their own hands, and in some way, it may work like a “self-fulfilling prophecy” (Merton 1948), influencing the effectiveness of external governance and co-governance, in turn.

More importantly, although there is always tension between the pursuits of legitimacy and profitability, they are not irreconcilable. In recent years, a concept called Corporate Social Innovation came to be on the rise, which claims that enterprises should abandon the traditional concept of Corporate Social Responsibility, which at most focuses on the short term in practical cases, and instead integrate the solution of social problems and the provision of innovative products and services into corporate activities as a technique for long-term growth, development, and prosperity (Dionisio and De Vargas 2020; Lu et al. 2020). However, as a modern bureaucratic organization, an enterprise’s decision-making also involves conflicts between its departments, and its complexity is no less than that of the government. It is also the reason why the actions of many enterprises are so “split apart” in internal governance.

Even if a technology is to be applied within an organization, legitimacy is necessary (Ren 2017). It is especially the case when pursuing good governance within the platform is the decisive element in all the decisions and development strategies of the enterprise. Although many corporate social responsibility or social innovation projects exhibit good governance performance, they are in an awkward position in the enterprise either because of low financial contributions or because the department itself is in a relatively marginal position in the corporate structure. In the past year or two, in the face of major public emergencies, some of these projects have been praised and promoted by relevant departments, and some have provided powerful weapons for enterprises to solve the problems of platform governance, which has enhanced the legitimacy of these projects. Thus, these received more resources from the company. However, the more powerful driving force comes from the changes in the international policy environment and social atmosphere. The pursuit of social values, promotion of public welfare, and strengthening of agent responsibilities have increasingly become a normal requirement for platform enterprises; through this connection, sociology can contribute to promoting good platform governance.

## Conclusions: social forces in the platform governance field and algorithmic governance as a sociological intervention

In addition to progress in economic efficiency and social benefits, the reason the platform economy could break through institutional constraints in the early years also had to do with its great number of beneficiaries, which expanded its social basis and enhanced its legitimacy (Zhang 2021). Metaphorically speaking, the platform is a “boat,” whereas society is the “water.” However, after years of expansion, the “boat” has become ever more powerful; its disorderly expansion has eroded society. Social forces can participate in platform governance through public opinion, user rights protection, and social crowdsourcing. Some platforms also maintain multilateral interaction on the app’s home page, which constitutes an important social mechanism to restrain platform technology from doing evil (Zhang and Hu 2021).

However, as measured against the advantages of the platform, social forces are still relatively weak. Many researchers have demonstrated workers’ resilience and even resistance in internet platform enterprises (Liang 2019; Sun 2019), but most of the time, they remain shackled. Others have deployed various tactics and strategies to avoid being “fed” by machines, platforms, and algorithms; they have even tried to change the output results of algorithms (Beer 2017). Nevertheless, these are the “weapons of the weak,” lacking social participation in general (Wang and Zhou 2019). In the face of this complicated situation, what contribution should sociology make to promote the participation of social forces in platform governance?

The article has emphasized that only by paying attention to the autonomy of platform companies, acknowledging the logic of these entities, and breaking the statist view that sees the platforms as passive “participants” or “executors” can we present and understand the holistic picture of platform government. Likewise, sociology also needs to consider the logic of the platform companies themselves to avoid a purely critical stand and contribute more constructive intervention to platform governance. This sort of sociological intervention should not only play an external role as a “social force” in co-governance at the “end” but also the “front” of platform self-governance, becoming an important link that monitors, evaluates, and promotes company awareness and ability to implement corresponding social values and initiatives. Sociological intervention can and should contribute systematic professional theory, knowledge, and techniques to realize the sustainable development of companies, build social platforms, and finally realize the compound optimization of social value and economic development.

The concept of sociological intervention is based on the “sociology of action”; instead of focusing on theoretical modeling and technical means (such as social prediction) to measure a stable society, it intends to reveal the oppression, domination, and inequality in the social structure, especially in the face of dramatic social changes, and participate in liberation with reflective actions (Shen 2008). Many applications of digital platforms have started to permeate the private and public lives of internet users from birth, which is the traditional category of sociological research. Sociological intervention can be used in platform governance in the following ways: algorithm governance, open-source platform empowerment, social value assessment, corporate social work, platform work participation, norm formulation, and training. Here, we want to emphasize the intervention method of algorithmic governance.

What we call “algorithm governance” is different from “algorithmic governance” (that is, government by algorithm) (Musiani 2013). The AI algorithm is the foundation for today’s digital platform companies to provide accurate services and expand their scales, online and offline. In terms of the business model and technological path used, these algorithms will inevitably create tension with privacy, security, and efficiency and thus become the origin of various crises. Algorithmic auditing is a vital part of algorithmic governance. It refers to the diagnosis and visualization of the unexpected consequences that should not have occurred in the algorithmic system, including but not limited to audit data manipulation, social bias, data deletion, social discrimination, invasion of privacy and property rights, and abuse of market ability and cognitive ability (Mittelstadt et al. 2016; Mittelstadt 2016; Sandvig et al. 2014).

Although the audit of program algorithms has existed for a long time, algorithm governance has its own characteristics in the era of AI.

From a technical point of view, AI can rapidly develop with the help of annotated data and prior knowledge, which are embodied in perceptual intelligence and cognitive intelligence, respectively. These aspects of intelligence correspond to connectionism, represented by machine learning, especially deep learning, symbolism, and logical representation and reasoning in cognitive schemas. In perceptual intelligence, machine learning and deep learning are often in a “black box” coupled with complex artificially adjusted parameters. It makes the final AI model inseparable from the subjective bias of all participants, including data collectors, data taggers, and programmers. Considering a simple example, we have an algorithm framework in which the facial data input only comes from a certain racial group. If the algorithm itself is a “clear code” and there is no deliberate bias after identification, it will still happen that the algorithm fails to apply to other racial groups. More than 40 kinds of similar subjective biases cannot be identified by auditing the “clear code.”

To take another example, as the basis of cognitive intelligence, a knowledge graph seems to be a neutral technical medium; however, the “knowledge” in it, either through the construction of vertical domain knowledge by the ontological approach or through the large-scale collection of lexical data on the network, is derived from the subjective construction of human beings, carrying different social prejudices and other risks. Thus, sociology is required to intervene in the closed loop of the process, especially at the source of the AI industry. The role of sociological intervention is to guide all parties to understand the consequences of algorithms as “social consequences” in algorithmic audits. After all, the algorithm of AI reconstructs social prejudice and discrimination through coding in the process of building a digital society. In other words, in addition to computer science, the algorithmic audit of AI needs sociology to provide systematic social reflection, professional theoretical judgment, and scientific methods of social research. The main approaches of the algorithmic audit include but are not limited to the following aspects.

The first is participating in data production and algorithm improvement at the source. Platform companies have the advantages of technological research & development and practical experience in AI and big data, which allow them to play an important role in the construction of digital governance systems on different levels, including the national level, be it the governance of data or data-based governance. However, this is not to say that technology can solve all problems (so-called “technological determinism”), nor that those platform companies are the main, or even the only driving force of social–technological progress as their marketing often claims (so-called “corporation determinism”) (Natale et al. 2019). Previous studies on big data found that despite occasional data-based analysis of social issues, many social scientists who want to research big data often encounter problems with no (social) data. Technology can provide tools and resources for data production, collation, and analysis. Nevertheless, sociological intervention is needed to determine what problems need more attention so that relevant data may be generated and collected. It is not difficult to understand that to study and solve social problems, the data we need should reflect the life and social conditions of different groups, by which we can construct statistical indicators and analytic models that provide evidence in investigations and studies of real-world inequality. The “data isolation” exposed in COVID-19 prevention and control also has something to do with this; so much attention was given to the IT infrastructure that we became oblivious to the weaknesses and hidden dangers of social governance, and as a result, “big data” failed to solve “small problems.” The same is true for algorithm optimization. In this regard, social prediction based on machine learning has made some pioneering attempts (Chen et al. 2020).

The second is to introduce a sociological perspective into content regulation. For example, gender inequality is a major problem in modern societies. However, according to our investigation of various search engines in China, gender bias and women’s rights in internet discourse have not received sufficient attention from major service providers. First, when typing keywords such as “woman,” “girl,” or “female” in the search box, most of the words and pictures in the top results are the results of stigmatizing (associated with search words such as “neurotic” and “rogue”) and consuming women (such as exposed bodies), which exposes obvious discrimination. Second, when searching for the words “doctor,” “nurse,” and “scientist,” the picture results only reflect a real occupational gender imbalance dominated by men, a form of hidden discrimination. Third, when searching for terms such as “domestic violence” and “mistreatment of women,” the algorithm fails to provide necessary help and support through technical means. As mentioned before, when AI merely repeats and even deepens inequality in the real world, it is not a good technology, contrary to the ethics of “AI for good.”

If we want to change the status quo, the first step should be to carry out the gender equality audit in AI and launch targeted interventions to prevent people from encountering increasing inequality in all aspects of life and work due to the misuse of technology. In a joint study with Baidu on AI social value, we posed this problem. After receiving a positive response, we cooperated with relevant departments to intervene with the algorithm of the search engine. In the initial stages of cooperation, we facilitated the weakening, shielding, and elimination of gender discrimination in search results. At the same time, we optimized and enhanced the functional channels to support women’s rights (Zhou and Lv 2022).^8^ The reasons for such positive results are that, on the one hand, Baidu has paid great attention to AI ethics and governance in terms of platform governance mechanisms, and on the other hand, the professional ability of sociology in the field of gender studies has contributed to the realization of this desire for change.

The third is participating in the construction of governance ecology in co-governance, empowering social communities to become “useful” actors. For example, in the “anti-COVID-19” campaigns, many companies launched functions such as mask detection and face recognition with masks to help prevent and control the epidemic and ensure the orderly resumption of work and school. However, many developers lack a full understanding of the ethical issues and the norms of using face recognition; they only provide a technical interface and skill training, ignoring the corresponding education of concepts and norms. From a corporate perspective, they will not invest a dime if they cannot understand and intuitively see what benefits social science will bring—economic performance, public legitimacy, or technological progress. On the other hand, the involvement of social scientists in the participation and building of digital governance has not been limited to abstract theoretical discussions; from very early on, they have flourished in empirical research, policy research, and behavioral intervention through corporate research institutes/think tanks, data opening plans, and various empirical research projects of their own choosing.

In recent years, China has made great progress in this area and has made outstanding achievements in some vertical areas, but it is still in the “catch-up” stage in general. Sociological intervention on this matter requires more solid empirical research results, more grounded action plans, and a more input-oriented understanding of penetration strategies to show, in a direct way, that “the increment of social science” can indeed make real contributions. In this way, social science researchers can become the real “fifth force,” in addition to government decision-makers, capital operators, technology developers, and the public media. More importantly, we need to cultivate more people capable of sociological intervention. On the one hand, sociologists should improve their digital literacy and skills and become architects, interpreters, and researchers in the “technological solutions of platform governance.” On the other hand, they should also empower and cultivate more people with different backgrounds to become developers who are literate in social science and computer science to promote “social solutions for platform governance.” Without the construction of social infrastructure, the application of fancy technology on the front end is limited and unsustainable.

In summary, the introduction of sociological intervention in all aspects of platform governance can better regulate the interaction between technology and society. It not only provides ideas and abilities to understand and solve social problems to digital platform companies interested in social innovation while maintaining their autonomy but also inhibits the possible damage to social interests through participation at the source, content audit, and social community empowerment so that they can play a positive role and promote the smooth implementation of technology.

More importantly, the sociological intervention into platform governance should be an indispensable application of computational sociology in practice. Computational sociology tends to emphasize the use of computational methods and complex statistical methods to excavate and integrate multiple forms of data to realize the description, interpretation, and prediction of complex social phenomena. However, in our view, social computing is not only a technical means but also a generative process for social reality. Just as computational sociologists can play an important role in developing new social computing tools, research on the relationship between computing and society and the social problems related to computing/technology should also be an important part of sociology. Although sociology has accumulated some experience, it has not yet fully possessed the ability and influence to make sociological interventions in the academic, disciplinary, and discursive system. How to make contributions to the “basic law of the digital era” should be something that sociology prepares for before facing the next “crisis.”

## Data Availability

We based this study on our data.

## Abbreviations

P2C: Platform-to-customer
P2B: Platform-to-business
P2G: Platform-to-government
AI: Artificial Intelligence
ESG: Environmental, social, and governance
CSR: Corporate social responsibility
NGOs: Non-governmental organizations

## Footnotes

1 Digitalization means turning nontraditional information sources such as text, image, and transaction records into digital form, thus providing more in-depth and data-driven insights for decision-makers. In today's world, digitalization is mainly driven by the so-called “platform.”

2 The digital platform is a “hybrid” entity. Some are operated by state-owned or private companies, some are state funded or operated institutions for e-government, and some are mixed (for example, through so-called public‒private partnerships). This article only discusses digital platform companies in the private sector. The reader can visit the websites of platform companies above.

3 Gillespie (2010; 2018) points out that the concept of “platform” is actually a metaphor or discourse deliberately created by internet companies, an excuse for what a neutral technology platform cannot do, thus hiding their responsibilities and escaping from traditional supervision; a “platform” is not a “plane,” but is composed of various heterogeneous communities with public responsibility.

4 Similarly, the external governance of the state on the platform companies will also affect other business and users in the platform’s economic network.

5 For various reasons, governments all over the world rarely take direct regulatory measures in reaction to some types of behaviors of platform users. Instead, they entrust the regulatory responsibility to platform companies themselves, who are responsible for what was traditionally the job of law enforcement and judicial institutions. The platform companies cooperate with the state to maintain the order of platform through risk control, compliance, and even specialized “platform governance” departments, which in fact is a kind of regulatory logic by which “the government regulates the platform; the platform regulates the subjects on the platform.” In the discourse system of China, this is called the “subject responsibility” of the platform, and now it is further promoted to the height of “primary responsibility.”

6 Different disciplines have different focuses; likewise, different scholars do not share the same concept of “platform governance.” It can refer to “governance of the platform” (platform as object, external governance), or “governance of the platform over other subjects (such as merchants on the platform)” (platform as subject, self-governance), or “governance on the platform” (platform as adverbial, co-governance).

7 District, city, province, and central government are different levels of the Chinese administration system.

8 For more details on this intervention, please see our website (www.baiji.org.cn). It is now disclosed in this anonymous version.
